# Functional characterization of chitin-binding lectin from *Solanum integrifolium* containing anti-fungal and insecticidal activities

**DOI:** 10.1186/s12870-017-1222-0

**Published:** 2018-01-03

**Authors:** Chang-Shan Chen, Chun-Yi Chen, Divya Malathy Ravinath, Agustina Bungahot, Chi-Ping Cheng, Ren-In You

**Affiliations:** 1 Department of Laboratory Medicine and Biotechnology, College of Medicine, Tzu Chi University, Hualien, Taiwan, Republic of China; 2 Institutes of Biomedical Sciences, Academia Sinica, Taipei, Taiwan, Republic of China; 3 Department of Life Science, Tzu Chi University, Hualien, Taiwan, Republic of China

**Keywords:** Chitin-binding lectin, *Solanum integrifolium*, Hemagglutination, Anti-fungal, Insect cells

## Abstract

**Background:**

Along with the rapid development of glycomic tools, the study of lectin–carbohydrate interactions has expanded, opening the way for applications in the fields of analytic, diagnostic, and drug delivery. Chitin-binding lectins (CBLs) play roles in immune defense against chitin-containing pathogens. CBLs from species of the *Solanaceae* family, such as tomato, potato and jimsonweed, display different binding specificities to sugar chains containing poly-N-acetyllactosamine.

**Results:**

In this report, CBLs from *Solanum integrifolium* were isolated by ion exchange chromatography. The fractions showed hemagglutination activity (HA). The recombinant CBL in the 293F cell culture supernatant was able to inhibit the growth of *Rhizoctonia solani* and *Colletotrichum gloeosporioide*. Furthermore, the carbohydrate-binding property of CBLs was confirmed with the inhibition of HA. Binding of CBL to *Spodoptera frugiperda* (sf21) insect cells can partly be inhibited by N-Acetylglucosamine (GlcNAc), which is related to decrease mitochondrial membrane potential of sf21 cells.

**Conclusions:**

The results showed that CBL exhibited antifungal properties and inhibited insect cell growth, which is directly correlated to the lectin-carbohydrate interaction. Further identification and characterization of CBLs will help to broaden their scope of application in plant defense and in biomedical applications.

**Electronic supplementary material:**

The online version of this article (10.1186/s12870-017-1222-0) contains supplementary material, which is available to authorized users.

## Background

Lectins are carbohydrate-binding proteins capable of binding polysaccharide and complex carbohydrates in a specific mode [1]. Lectins react with sugar ligands in glycolipid, glycoprotein or oligosaccharide formats. Thus they have been recognized as useful tools for many applications such as agglutination, anti-tumor treatment, immunomodulation [2, 3], and inhibition of the growth rate of insects, fungi, bacteria, and viruses [4, 5]. The plant lectins isolated from *Erythrina velutina* specifically interact with A, B, and O blood groups whereas lectins from *Calpumea aurea*, *Dolichos biflorus* and *Sophora japonica* have the ability to bind to A and B blood groups. Other non- A, B and O blood groups also can be differentiated by plant lectins, for example, lectin isolated from *Iris amara* shows specificity to the M blood group and those from *Bauhinia purpurea* and *Vicia graminae* lectins show specificity to the N blood group [6, 7]. The ability of plant lectins to agglutinate different types of blood cells are due to prefer recognition of the specific glycan patterns. There are twelve families of plant lectins which can be categorized into Amaranthin, *Agaricus bisporus* agglutinin, chitinase-related aggutinin, Cyanovirin, *Euonymus europaeus* agglutinin, *Galanthus nivalis* agglutinin, Hevein domains containing protein, Jacalins-related lectin, legume lectin, the LysM motif, Nictaba, and the Ricin-B family based on the structural similarity of carbohydrate recognition domain (CRD) [8]. Most lectins bind to unique exogenous glycan patterns but not to endogenous (self-produced) glycans. The specific recognition of exogenous carbohydrate structures gives evidence that they might have roles in creating defense systems. Indeed, some plant lectins are induced during stress and display defensive characters to increase insecticidal, bactericidal, and antifungal activities [9, 10].

Many plants and animals have chitin-binding lectins (CBLs) for their immune defense to against chitin-containing pathogens. Chitin is an abundant biopolymer in nature which is assembled with GlcNAc repeated units, widely distributed within exoskeletons of insects, cell walls of fungi, eggs of nematodes, marine diatoms, and shells of crustaceans and zooplankton. The GlcNAc repeated units are linked by glycosidic bonds from the homopolymer of chitin [11]. Many plant CBLs have common structural motifs which are composed of cysteine-rich amino acid sequences called chitin-binding domains (CBDs) [12]. Some plant CBLs contain multiple CBDs. For example, *Urtica dioica* agglutinins (UDA) from *Urtica dioica* and *Solanum tuberosum* Lectin (STL) from potatoes have two CBDs, whereas wheat germ agglutinins (WGA) from wheat germ and *Lycopersion Esculentum* Lectin (LEL) from tomatoes have four CBDs [13]. Moreover, CBLs from species of the *Solanaceae* family do not only have CBDs but also have an additional hydroxyproline (Hyp)-rich domain, for example, jimsonweed (*Datura stramonium*; DSA), tomato (*Solanum lycopersicum*; LEL), potato (*S. tuberosum*; STL) and hold an additional Hyp-rich domain similar to the cell wall glycoprotein extensin which is applied to incorporate glycosylation motifs [14]. Although the HA of these CBLs can be similarly inhibited by chitin, the preference of the glycan-binding specificities to glycoproteins and sugar chains are dissimilar. There are many glycomic applications based on different lectins for identifying new biomarkers or treatments for different diseases [15, 16]. Thus, it is imperative to explore more kinds of chitin-binding lectins to increase in the lectin microarray platform; this may improve the sensitivity and discrepancy of glycans with GlcNAc moieties.

Scarlet eggplant (*Solanum integrifolium Poir.*) belongs to the *Solanaceae* family, and is also known as bitter tomato. It is documented as an indigenous medicinal vegetable consumed by Taiwanese aborigines for their anti-inflammatory effects [17, 18]. However, whether *S. integrifolium* contains CBLs resembling those of other species from *Solanaceae* family is not known. In the present study, we have isolated CBLs from *S. integrifolium* using chromatography, and evaluated biological properties of CBLs in vitro. The molecular characterization of CBLs from *S. integrifolium* allows us to compare their unique identity with those of other species of *Solanaceae* family for future applications.

## Results

### Purification of lectin from *Solanum integrifolium*

**Fig. 1:**
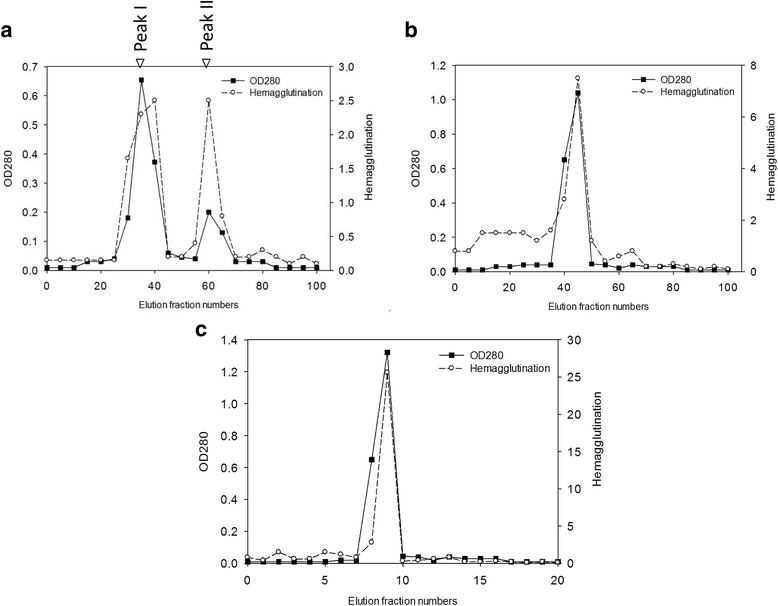
Purification of eggplant lectin. **a** Ion exchange chromatography of the eggplant crude extract on a DEAE Sephadex A-25 column (1.0 cm × 20 cm). The column was pre-equilibrated with 0.02 M Tris-HCl buffer, pH 8. The bound protein was eluted using a 0.02–1 M NaCl gradient in 5 ml fractions at a flow rate of 0.5 ml/min and the fractions were monitored at 280 nm. A hemagglutination titer was assayed by red blood cells (RBCs) agglutination using trypsinized human blood cells. **b** Fractionation of peak I from the ion exchange column on a Sephadex G-75 column (2.6 cm × 40 cm) with 0.15 M NaCl in 0.02 M Tris-HCl (pH 8.0). The protein was eluted using the same buffer in 3 ml fractions at a flow rate of 0.5 ml/min. **c** Chitin gel column chromatography (1.0 cm × 20 cm) of the proteins eluted from Sephadex G-75 column. The bound protein was eluted using 20 mM acetic acid in 0.5 ml fractions at a flow rate of 0.1 ml/min

According to previous reports, species of the genus *Solanum* contain chitin-binding lectins, which play a defensive role in plants. In order to purify CBL from *S. integrifolium* (scarlet eggplant), extracts from eggplant fruits were immobilized using DEAE Sephadex A-25 and Sephadex G-75 column chromatography. There are two peaks showing HA gained from Sephadex A-25 chromatography (Fig. 1a). The concentrated peak I fraction was further subjected to a Sephadex G-75 column which also showed a major HA activation peak (Fig. 1b). To gain further information on the chitin-binding property of CBL, the fraction with highest HA activity was loaded to chitin resin beads. The bounded protein in chitin beads, when eluted with binding buffer, showed a single peak with HA (Fig. 1c).

### Characterization of eggplant lectin

**Fig. 2:**
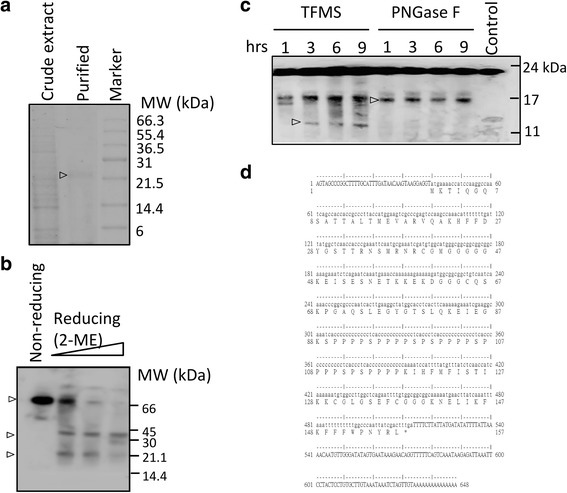
Determination of lectin purified from eggplant. **a** Results of SDS-PAGE analysis of crude extracts and eluted proteins with chitin bead purification. **b** The reaction mixtures with 2-ME (0, 3, 10, and 30%) were analyzed by non-reducing SDS-PAGE and stained with Coomassie Brilliant Blue. **c** Deglycosylation of purified lectin. Purified CBL was treated with or without trifluoromethanesulfonic acid (TFMS) and peptide-N-glycosidase F (PNGase F) and then analyzed by SDS-PAGE. The arrow indicates deglycosylation form. **d** Amino acid sequence obtained from matrix-assisted laser desorption ionization (MALDI) mass spectrometry. Comparison nucleotide sequence with deduced amino acid sequence by EMBOSS prettyseq (http://www.bioinformatics.nl/cgi-bin/emboss/prettyseq)

Affinity-purified lectin fractions were concentrated and simultaneously subjected to 15% SDS-PAGE and native PAGE. Two major bands were identified by SDS-PAGE with a molecular weight of 30 and 20 kDa (Fig. 2a, lane 1), respectively, while in non-reducing SDS-PAGE, CBL molecular size was about 66 kDa (Fig. 2b). The protein was affected by the presence of reductant 2- mercaptoethanol (2-ME), suggesting that CBLs contain disulfide bonds (Fig. 2b, lanes 2 to 4). The results indicate that the oligomeric forms of CBL observed under non-reducing conditions.

Protein glycosylation is a frequent post-translational modification which apparently increases protein molecular weight. We examined the glycosylation state of the CBL by in vitro enzymatic digestion to determine the accurate size of the protein moiety. The deglycosylated CBL was generated from trifluoromethanesulfonic acid (TFMS) and peptide-N-glycosidase F (PNGase F) incubation. Using TFMS to digest glycosidic linkages can result in O-linked oligosaccharides and N-linked oligosaccharides being removed from CBLs, and without digesting peptide bonds. We found that TFMS-treated CBLs became smaller than their untreated forms. (Fig. 2c, lanes 1 to 4). N-glycans released by N-glycosidase F (PNGase F) also resulted in a smaller protein band compare to the control (Fig. 2c). To obtain the amino acid sequence information of purified lectin, fractions corresponding to the putative lectin were cut out from the SDS-PAGE and N-terminal peptide sequencing was performed followed by mass spectrometry. We identified the N-terminal amino acid sequence of fractionated CBL as MKTIQGQSATTALTMEVARVQA. To obtain more information of peptide sequence, additional sequencing was determined from tryptic digested peptide fragments. Mass spectrometry analysis of the tryptic in-gel digests identified the unique sequences (Additional file 1: Table S1).

We designed the degenerate primers from N-terminal sequence and other primers from a partial sequence (Additional file 1: Table S2). The full-length cDNA sequence of CBL was further acquired using the PCR method in various combinations of primers. Sense and anti-sense degenerate primers were referenced from the tryptic digested peptide from mass spectrometry. The deduced amino acid sequence of the open reading frame (471 bp) of CBL is composed of 157 amino acids (Fig. 2d), which contain the peptide sequences obtained from an internal amino acid analysis using trypsin. The isoelectric point (pI) of CBL was estimated to be 9.46 and the molecular mass is 16,812.18 Da.

### Molecular property of recombinant scarlet eggplant CBL

**Fig. 3:**
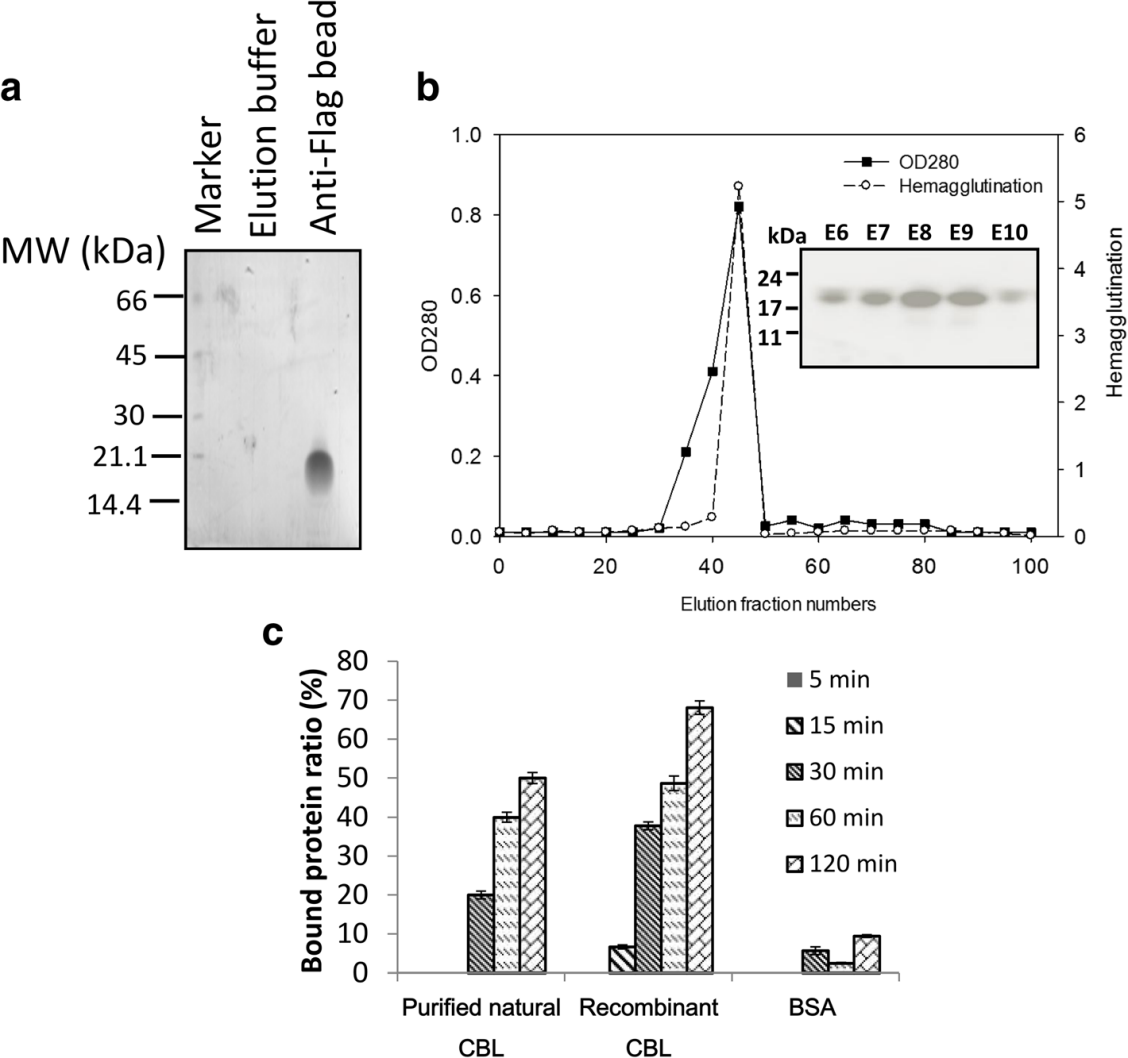
Molecular properties of recombinant CBL. **a** Coomassie brilliant blue staining of the recombinant CBL purified using anti-Flag bead from the 293F culture supernatant. **b** Chitin gel column chromatography of the recombinant CBL purified from 293F culture supernatant. The bound protein was eluted using 20 mM of acetic acid in 0.5-ml fractions at a flow rate of 0.1 ml/min and the fractions were monitored at 280 nm. The hemagglutination titer was assayed by RBC agglutination using trypsinized human erythrocytes. The eluted proteins were subjected to glycol chitin containing gel and Calcofluor White M2R staining was performed. **c** Comparison of chitin-binding activity. The purified natural CBL and recombinant CBL were incubated with chitin for indicated time intervals at 37 °C. The supernatant was evaluated by spectrophotometry to perform the residue-unbound CBL. The ratio of binding was calculated by OD ratio of unbound from control to CBL serial concentration groups. BSA is used as negative control

The cDNA isolated from CBL was cloned into the 293F expression system to produce soluble recombinant lectin. The lectin level from the culture supernatant that was assessed to contain 1.6 mg per liter of purification (Fig. 3a). The chitin-binding specificity of recombinant CBL was evaluated by chitin affinity column chromatography and HA activity similar with Fig. 1c. The inhibition of HA of sugars and sugar derivatives were performed. The results demonstrated specifically competition effect of GlcNAc to CBL at the minimum inhibitory concentration of 3.12 mM (Table 1), which suggested that CBL had glycan binding specificity. To analyze the activity of CBL in different conditions of temperature, pH and metal ions, HA was evaluated in temperature range of 50 to 75 °C, pH range from 0 to 14 and divalent cations such as CaCl_2_, CuCl_2_, FeCl_3_, MgCl_2_, MnCl_2_, and ZnCl_2_. The activity of CBL was heat stable in the temperature range of 50 to 65 °C with optimal activity at 50 °C (Additional file 2: Figure S2). In a pH range of 4 to 8, the activity was stable and showed a maximum activity at pH 6. At pH 2 and 9, protein remained with 20% lectin activity; rapidly fell to only 1% residual activity with pH 12–14 buffer (Additional file 2: Figure S3). In the presence of metal ions, little effect of metal ions on activity of CBL was found (Additional file 1: Table S3). Moreover, the recombinant CBL was subjected to SDS-PAGE, with glycol chitin added as the substrate. After gel electrophoresis, the undigested glycol chitin in gel was stained with calcofluor, which showed a dark band against the UV fluorescence (elution 6 to elution 10: E6~E10, Fig. 3b). The eluted fractions from chitin gel column containing HA (E6~E10) were collected for chitin binding assay. Furthermore, the chitin binding of CBL was performed by co-incubation of chitin with purified natural CBL, recombinant CBL and bovine serum albumin (BSA; control). The unbound proteins were analyzed by a spectrophotometer. We found the bound protein in CBL groups were increased with time dependent manner, however, the level was not changed in the BSA group (Fig. 3c). This suggested that CBLs but not BSA specifically bind to chitin.

**Table 1:** Inhibition of hemagglutination by CBL with sugars and sugar derivatives

Sugars	^a^MIC (mM)
D-glucose	^b^No inhibition
D-fucose	^b^No inhibition
D-mannose	^b^No inhibition
D-glucosamine	100
D-xylose	^b^No inhibition
GlcNAc	3.12
GalNAc	25
ManNAc	^b^No inhibition
Sucrose	^b^No inhibition
Lactose	^b^No inhibition
Mannan	^b^No inhibition

^a^Minimum inhibitory concentration (mM): The lowest concentration of sugars that inhibited HA of CBL were represented

^b^No inhibition: Sugars and sugar derivatives showed no inhibition of CBL activity up to concentrations of 200 mM

### Antifungal activity of CBL

**Fig. 4:**
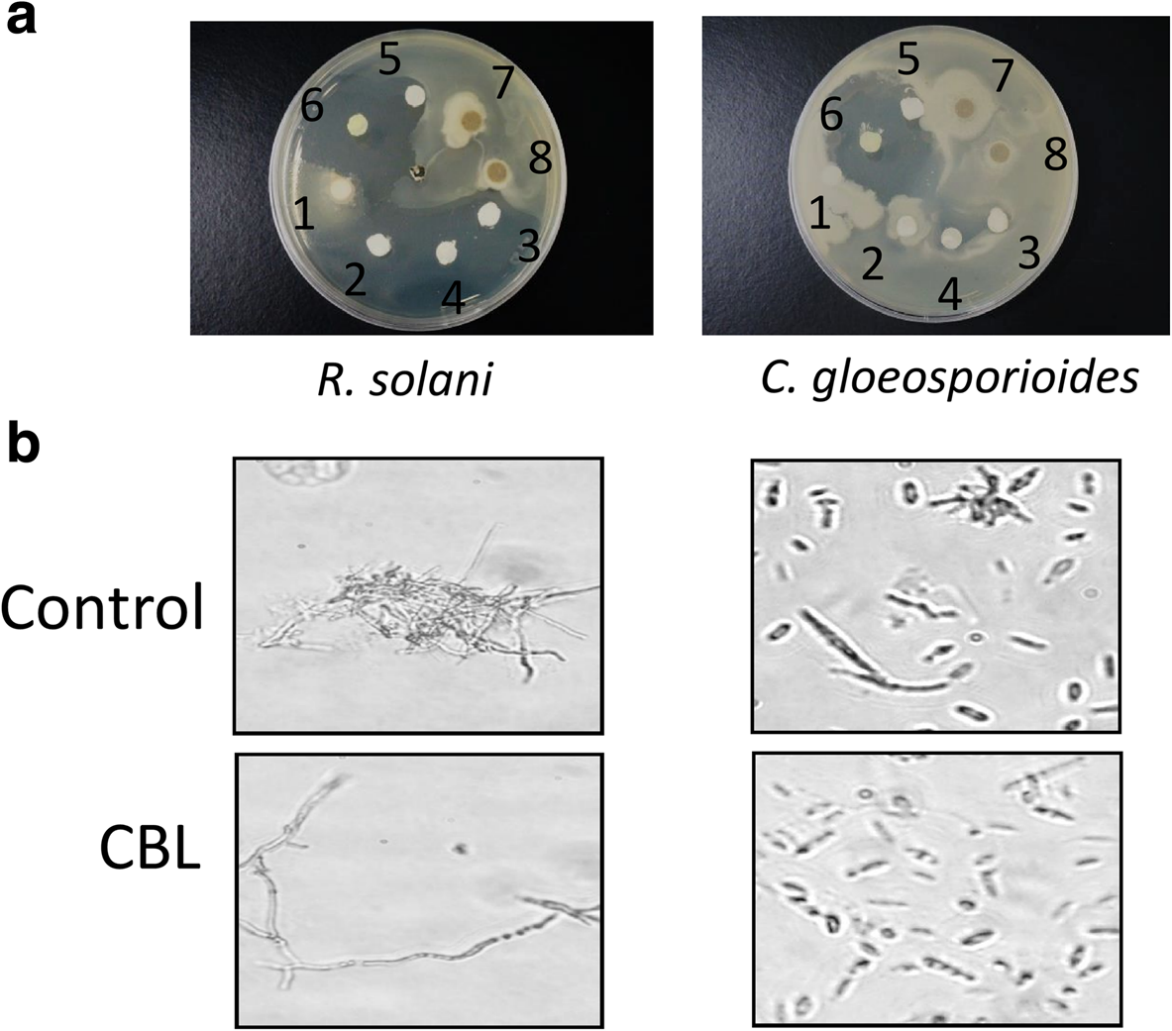
Effect of CBL on fungus. **a** In vitro growth inhibitory activity of CBL against *Rhizoctonia solani* and *Colletotrichum gloeosporioides* in plate assay. Disc contain (1, 2) crude extracts 1 and 5 mg/mL; (3, 4) purified natural CBL 1 and 5 mg/mL; (5, 6) recombinant CBL 1 and 5 mg/mL; (7, 8) boiled CBL 1 and 5 mg/mL. **b** Evaluation of CBL activity in liquid assays. CBL (100 μg/mL) was treated into the potato dextrose broth that containing with *R. solani* and *C. gloeosporioides* at 25 °C for 48 h. Inhibition rate of the growth of fungal hyphae was investigated daily

To assess antifungal activity, both plate and liquid assay were performed to evaluate fungal growth. The *Rhizoctonia solani* and *Colletotrichum gloeosporioides* were incubated with and without various concentration of the crude extract, purified CBL or recombinant CBL of *S. integrifolium* (Fig. 4a). Both purified and recombinant CBLs presented growth inhibition of fungus in a plate assay. The recombinant CBL showed inhibition of growth against with a zone inhibition diameter of 8 mm and 12 mm in *C. gloeosporioides* and *R. solani* respectively (Additional file 1: Table S4. After incubation, less growth of *R. solani* was observed in the recombinant CBL treatment (Fig. 4b). *C. gloeosporioides* did not show efficient growth inhibition compared to *R. solani* (Fig. 4b).

### CBL causes reduced mitochondrial membrane potential in sf21 insect cells

**Fig. 5:**
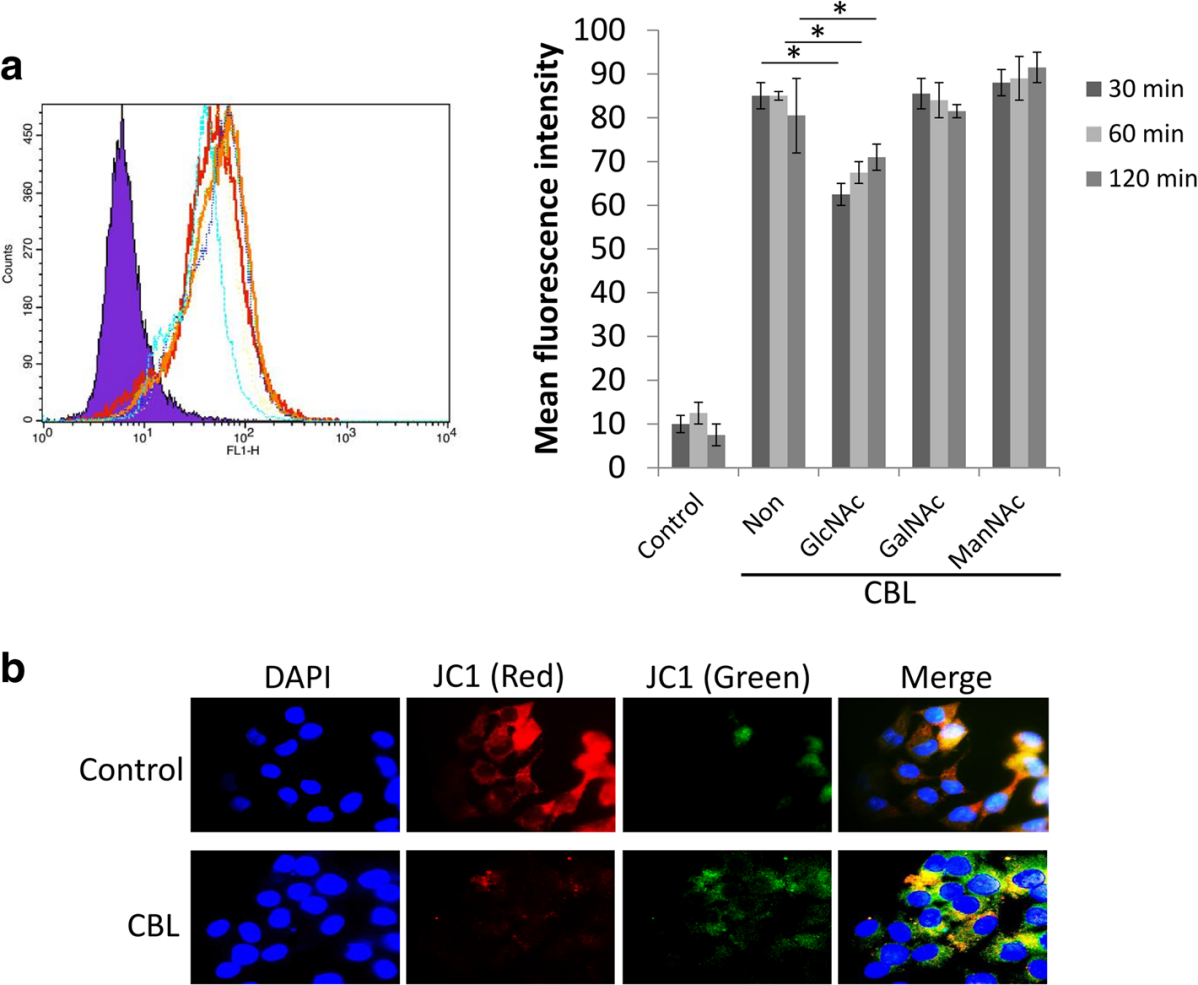
Effect of CBL on insect cells. **a** CBL-FITC binding to sf21 cells. CBL-FITC (1μg/mL) was added to sf21 cells for 30 mins. The surface binding of CBL-FITC to sf21 cells was determined by mean fluorescence intensity (FL1) using flow cytometry. The sugar used as a competitor in the binding solution of CBL-FITC and sf21 cells was indicated. Purple: cells without CBL-FITC treatment; light blue: N-Acetyl-D-glucosamine pretreated (GlcNAc); red: N-Acetyl-D-galactosamine pretreated (GalNAc); deep blue: CBL-FITC alone (Non); orange: N-Acetyl-β-D-mannosamine pretreated (ManNAc). **b** CBL reduces the mitochondrial membrane potential in sf21 cells. Sf21 cells were incubated with or without CBL for 24 h. Cells were stained for JC-1 to measure the change in mitochondrial membrane potential and observed under the fluorescence microscope. The reduction in mitochondrial membrane potential corresponds to increase in green fluorescence (free JC-1) and decrease in red fluorescence (aggregates JC-1)

When we tried to express CBLs in the sf21 insect cells expression system, the CBL construct-transfected sf21 cells had fewer alive cells and it was hard to generate baculovirus for protein amplification (data not shown). To assess the possibility of CBL affecting the sf21 insect cells growth condition, we examine the interaction of CBL with sf21 cells by fluorescein isothiocyanate (FITC)-labeled CBL binding assay. The recombinant CBL purified from 293F cell culture supernatant was labeled with FITC and incubated with sf21 cells. Flow cytometric data demonstrated the presence of CBL-FITC on sf21 cells. To further verify the binding specificity of the lectin-carbohydrate interaction, GlcNAc, GalNAc and ManNAc competitors were added into CBL-FITC and sf21 cells binding solution. The binding affinity of CBL-FITC on the sf21 cell surface was decreased in the GlcNAc treated group (Fig. 5a). To further confirm CBL effect on sf21 cells, the mitochondrial function following CBL stimulation was demonstrated by 5,5′,6,6′-tetrachloro-1,1′,3,’-tetraethylbenzimidazolylcarbocyanine iodide (JC-1) staining. A predominant red fluorescence in control group indicated an aggregated JC-1 form in mitochondrial membranes (Fig. 5b, upper panel), whereas green fluorescence enhanced in CBL-treated sf21 cells reflects the presence of free JC-1, indicating the depolarized mitochondrial membrane potential (Fig. 5b).

## Discussion

The present study analyzed whether CBL from *S. integrifolium* possessed anti-fungal activity towards two types of fungal plant pathogens *R. solani* and *C. gloeosporioides*. Early study has identified plant lectin UDA from *Urtica dioica* inhibited phytopathogenic and saprophytic fungi growth in vitro [19]. Later on, hevein, a chitin-binding protein from the rubber-tree (*Hevea brasiliensis*) was also reported as a potent inhibitor for several fungi [20]. In addition, potato [21], *Setcreasea purpurea*, Chinese herb *Astragalus mongholicus* [22] and jackfruit [23] contain chitin-binding lectins that have anti-fungal activity. Recently, the lectin of Bangladeshi cultivar, ‘Deshi’, of potato (*Solanum tuberosum L.*) containing a glycan-binding property with an affinity to GlcNAc polymers was identified. The first observation was made that potato lectin prevented biofilm formation by *Pseudomonas aeruginosa* [24]. The chitin-binding property of CBL suggested its relationship in affecting not just the fungal cell wall structure, but also bacteria biofilm. These findings support the potent role of plant lectins in defense and applicable for preventing infection.

Chitin (C8H13O5N)n is natural polysaccharide which is composed of GlcNAc through a β, 1–4-linked polymer. Lectins from species of the *Solanaceae* family, such as tomato, potato and jimsonweed contain chitin-binding domain (CBD) [25], which is an activation site for catalyzing and hydrolyzing the glycosidic bonds of chitin [26, 27]. Besides, they hold an additional hydroxyproline-rich domain that resembles the cell-wall glycoprotein extensin which is applied to incorporate characteristic β-L-arabinofuranoside repetitive glycosylation motifs [14]. Our study identified CBL from *S. integrifolium*. The amino acid sequence of CBL is unique among chitin-binding lectins from other species of *Solanaceae* family (Additional file 2: Figure S1) [28, 29]. It contains PPPPS repeats which resemble LEL, STL and DSA chitin binding lectins. The similarity of RCGMGGGXGKXXXXSNE motif from CBL compared to tomato and potato lectins also can be found near N-terminal.

Generally, the plant lectins are purified from their natural sources, such as germs, leafs, seeds, fruits, tubers and roots. Sometimes, the purification process is time-consuming and presenting isoforms and impurities in each batch. At present, different expression platforms have been developed for recombinant lectin production which make the investigation of the biological activity of the lectins more feasible [30]. In this work, CBL can be properly produced in the 293F expression system and the recombinant product was expressed into the culture medium. The recombinant CBL after chromatography purification still has the HA activity and the anti-fungal activity further ensures possible application of 293F expression platforms for recombinant plant lectins production. Moreover, we found that limited expression of CBLs in sf21 insect cells platform is due to CBLs binding to insect cells and decreasing the mitochondrial membrane potential of the sf21 cells themselves. These results suggest that CBLs affect sf21 insect cell growth conditions via carbohydrate recognition and depolarized mitochondrial membrane potential. In the future, engineering the insect cell N-glycan processing pathway [31], based on rapid assays for lectin binding changes [32] and lectin toxicity induced apoptosis [33] will rapidly characterize the glycan recognition and function of lectins. These data give information for glycomic profiling and will help applications of glycomic analysis and technique development, and may be useful as particularly for biomedical therapeutic applications [34].

## Conclusion

Here, we identify novel CBL from *S. integrifolium* which can be purified by ion-exchange chromatography and chitin affinity column chromatography. The recombinant CBL produced from 293F cell culture supernatant maintained their carbohydrate-binding specificity. The carbohydrate-binding property of CBL confirmed that binding to sf21 insect cells can partly inhibit by GlcNAc, but not GalNAc or N-Acetylmannosamine (ManNAc). Furthermore, addition of recombinant CBL to *R. solani* and *C. gloeosporioides* culture impeded their growth, proving that CBL binds to chitin.

## Methods

### Materials

The *S. integrifolium* fruits were purchased from food markets in Hualien, Taiwan. These samples were stored in labeled ziploc plastics bags in a − 80 °C refrigerator for lectin purification. Sephadex A-25, Sephadex G-75 and other reagents were acquired from Sigma-Aldrich.

### Preparation of crude extract

The extraction protocols were modified from a previously published procedure [14]. Briefly, the fresh fruits were cut into pieces and dried in an oven at 50 °C, then mixed with 0.1 M NaCl at 4 °C for 48 h. After filtration by mesh (80 um, BD Falcon) and centrifugation at 12000 rpm for 30 min at 4 °C, the supernatant was subjected to ammonium sulfate (40~70%) precipitation. The pellets were dissolved in distilled water and dialyzed in water overnight at 4 °C.

### Purification of lectin

The extract from *S. integrifolium* was subjected to DEAD Sephadex A-25 column (1.0 cm × 20 cm). The column was washed with dialysis buffer (0.1 M NaCl in 0.02 M Tris-HCl, pH 8.0) and a linear gradient of 0.02 to 1 M NaCl (flow rate 0.5 ml/min) purification was performed. The elution fractions were subjected to hemagglutination assay. Those fractions with hemagglutination activity were further applied to a Sephadex G-75 column (2.6 cm × 40 cm). The fractions containing hemagglutination activity were concentrated and dialyzed against phosphate-buffered saline (PBS) using an Amicon® Pro Purification System with a YM-10 membrane (Millipore Co., Billerica, MA, USA). The concentrated proteins eluted from Sephadex G-75 column were diluted into chitin gel binding buffer (50 mM Tris HCl, 1 mM EDTA, 500 mM NaCl, 0.1% Tween-20, pH 8) and loaded in a chitin gel column (New England Biolabs) equilibrated with binding buffer. After washing column, bound proteins were eluted with 20 mM acetic acid, and dialyzed in 5 mM sodium phosphate buffer, at pH 6.0. The fractions from chromatography were stored in −80 °C for electrophoresis.

### Hemagglutination assay

The HA of lectin was performed in round-bottomed 96-well plates (Corning) using a panel of type A1, A2, B, and O obtained from Formosa Biomedical Technology Co (Taiwan). Trypsinized erythrocytes were washed with 0.05 M Tris-HCl, pH 7.5 and centrifuged at 3000 rpm for 5 min at room temperature. Erythrocytes were diluted in phosphate-buffer saline (PBS, pH 7.2) to a 4% concentration. The 4% suspension of washed RBCs (50 μl) were mixed with a serial dilution of lectin that was purified from *S. integrifolium* extracts in PBS, (pH 7.2). The specific haemagglutinating unit was defined as the minimum amount of lectin mixtures (mg) that agglutinated RBCs in suspension after 60 mins.

### Electrophoresis of purified lectin

The purified lectin was subjected to SDS-PAGE and native PAGE. The concentrated protein was diluted in 5× sampling buffer (312 mM Tris-HCl, pH 6.8, 10% SDS, 50% glycerol, 25% 2-mercaptoethanol and 0.01% bromophenol blue). For non-reducing protein analysis, protein fractions were mixed with 5× sampling buffer with or without serially diluted 2-mercaptoethanol. Then, samples were separated by gel electrophoresis, stained with Coomassie brilliant blue and destained with 10% acetic acid.

### Deglycosylation assay

Trifluoromethanesulfonate (TFMS; Sigma) was used to remove both N- and O-glycans from glycoproteins. Concentrated proteins were treated with precooled, anhydrous TFMS on ice. At the end of the digestion, samples were neutralized with pyridine–methanol–water solution (pyridine-methanol-water [3:1:1]). N-Glycosylation was removed using PNGase F (Sigma-Aldrich, United States) according to the manufacturer’s manual. Concentrated proteins were mixed with PNGase F in 50 mM of sodium phosphate buffer, pH 7.2 at 37 °C. The mixtures were separated on SDS-PAGE and gels were stained for carbohydrate with periodic acid-Schiff (PAS) stain. The samples treated without the addition of deglycosylation reagents served as negative control.

### Amino acid sequence analysis

Protein bands were subjected to SDS-PAGE and target spots were excised from gels followed by trypsin digestion at 37 °C overnight. N-terminal amino acid sequencing was determined by Edman degradation. The eluted protein was deglycosylated by PNGase F and further digested with chymotrypsin gold (Promega, Uinted States) overnight. For protein identification, the digested peptide mixtures were forwarded to matrix-assisted laser desorption ionization mass spectrometry (MALDI-MS). The results were analyzed by MASCOT (Matrix Science). Multiple alignments were performed using MAFFT (http://www.ebi.ac.uk/Tools/msa/mafft/) under default settings (gaps and matches were equally weighted).

### Recombinant CBL production

For cloning of the scarlet eggplant lectin and plasmid construction for protein expression, total RNA of *Solanum integrifolium* was harvested from the fruit part of scarlet eggplant and re-suspended in Trizol reagent (Invitrogen). To synthesize the first-strand cDNA, the total RNA was isolated using total RNA Mini kit (Geneaid, Taiwan) and reverse transcription was conducted using Deoxy + HiSpec Reverse transcriptase (Yeastern Biotech Co., Taiwan) according to the manufacturer’s instructions. PCR was performed to amplify cDNA using different combinations of degenerated primers (Additioanl file 1: Table S2) designed based on amino acid sequences obtained from mass spectrometry. The PCR products were subjected to agarose gel electrophoresis, conducted gel elution (Qiagen), and DNA sequencing (Mission Biotech Co., Taiwan). The DNA sequencing results of PCR products were analyzed by EMBOSS prettyseq (http://www.bioinformatics.nl/cgi-bin/emboss/prettyseq) and compared with MS data.

The PCR product of CBL full length coding sequence was sub-cloned into pCMV-Flag plasmid (Sigma). The plasmid-contained CBL construct was transiently transfected using 293 Fectin (Invitrogen) into 293F cells (kindly provided by Dr. Shie-Liang Hsieh, Academia Sinica, Taiwan), cultured in 293 Freestyle media (Life Technologies) and grown for 96–144 h. The culture supernatants were purified using anti-FLAG beads according to the manufacturer’s protocol (Sigma-Aldrich).

### Chitin binding assays

To compare binding efficacy of the purified natural CBL and recombinant CBL, the chitin binding assay was performed. The serial dilutions of CBL were incubated with chitin (Sigma) from 5 mins to 2 h at 37 °C. After a brief centrifugation (10,000 x g, 3 mins), the unbound CBLs were shown at supernatant and CBL-bonded chitins were formed in pellets. The supernatant was evaluated by spectrophotometer (Multiskan Spectrum, Thermo Scientific) to analyze the residue-unbound CBL. The ratio of binding was calculated by optical density (OD) ratio of unbound from control to CBL serial concentration groups. BSA is used as negative control.

### Effect of temperature, pH and metal ions

To test the activity of CBL in different conditions of temperature, pH and metal ion concentrations, purified lectin were mixed and incubated with different pH or different metal ion solution for indicated time intervals. The following reagent stocks were prepared to modify pH from 0 to 14: 1 M HCl, 1 M NH_4_OAc, 1 M Tris-HCl, 1 M NaHCO_3_ and 0.1 M NaOH. To test the effect of temperature, purified lectin was exposed at 50 °C to 75 °C for indicated time intervals. The mixtures were cooled on ice immediately and then warmed to room temperature for the HA activity assay. To examine the effect of metal ions on CBL, purified proteins were dialyzed in 50 mM EDTA buffer to remove metal ions. The reaction mixtures were concentrated and exchanged into 50 mM Tris-HCl buffer, pH 7.2. The metal ion solution was prepared in the presence of 50 mM chloride salts of Ca, Cu, Fe, Mg, Mn, Zn or EDTA.

### Hemagglutination inhibition assay

To investigate the specificity of carbohydrates on CBL activity, the inhibition of HA by sugars was determined in round-bottomed 96-well microtiter plates. Various sugars and sugar derivatives were serially diluted with PBS. The lectin samples were incubated with the same volume of serial dilution of sugars for 10 min at room temperature (RT) before a 4% RBCs suspension was added to each well. The concentration of the sugars that showed complete inhibition of HA after 1 h was taken as the minimum inhibitor concentration (MIC). The results reported are the mean of three determinations.

### Antifungal assay

To evaluate the antifungal activity of CBL, disc diffusion and liquid assay were performed. The spores of *Rhizoctonia solani* and *Colletotrichum gloeosporioides* were cultured into Petri dishes containing potato dextrose agar (PDA). The concentrated CBL (1 and 5 mg/mL) from crude extract, purified, recombinant or boiled conditions were applied to sterilized paper discs (5 mm in diameter) using a capillary pipette. The degree of growth inhibition was measured in diameter (mm) of non-growth zone. A liquid assay system was also used by adding CBL (100 μg/mL) into the potato dextrose broth that containing *R. solani* and *C. gloeosporioides* (Protoplast: 10^6^/100 μL). The boiled CBL was used as a negative control. The mixtures were cultured at 25 °C for 48 h. The growth of fungal hyphae was examined daily under microscope.

### Measurement of CBL binding by flow cytometry

CBL proteins were labeled with FITC (Sigma). Briefly, FITC was added to CBL in 0.1 MNaHCO_3_ (pH 7) for 30 min at 4 °C. The mixture of FITC with CBL was separated by chromatography on a PD-10 desalting column (GE Amersham) to remove free FITC. The eluted CBL-FITC fractions were concentrated in Amicon Ultra Centrifugal filter (Sigma) and protein concentration calculation was calculated using the absorbance at 280 and 490 nm. For binding assay, *Spodoptera frugiperda* (Sf) 21 cells (kindly provided by Shr-Jeng Jim Leu, National Yang-Ming University, Taiwan) were cultured in Grace’s Insect medium and seeded onto a culture plate (6-well, Costar) overnight. CBL-FITC (1 μg/mL) was added to sf21 cells for 30 mins. To compete the lectin–carbohydrate interaction, the indicated sugars and sugar derivatives (50 mM) were used as competitors in CBL-FITC and sf21 cells binding solution. The stained cells were washed twice with cold PBS and analyzed in a Gallios flow cytometry using Kaluza software (Beckman).

### Measurement of mitochondrial membrane potential

To examine the mitochondria membrane potential, JC-1 staining was performed. Sf21cells (5 × 10^5^) were incubated with CBLs (5 μg/mL) for 24 h. The cells were washed with PBS and centrifuged at 2000×g for 5 min. After washing with PBS twice, the cells were stained with 2.5 μg/ml JC-1 dye at 37 °C in the dark. The mitochondrial patterns were analyzed using a fluorescence microscopy.

## Additional files

Additional file 1: Table S1. Tryptic digested peptide sequences identified by MS. **Table S2.** Primer sequences used in this study. Table S3. The activity of CBL in various metal ions. **Table S4.** In vitro inhibition effect of CBL on fungus. (PDF 120 kb)

Additional file 2: Figure S1. The multiple alignment of LEL, STL and DSA to CBL. Multiple alignments were performed using MAFFT (http://www.ebi.ac.uk/Tools/msa/mafft/) under default settings (gaps and matches were equally weighted). **Figure S2.** The effect of temperature on CBL. The lectin was preheated with 50, 60, 65, 70, or 75 °C for different durations. **Figure S3.** The effect of pH on CBL. The lectin was incubated in the solutions of pH 0 to 14 at RT. Results are representative of >3 independent experiments. (PDF 341 kb)

## Abbreviations

CBDs: Chitin-binding domains
CBLs: Chitin-binding lectins
FITC: Fluorescein isothiocyanate ()
GlcNAc: N-Acetylglucosamine
HA: Hemagglutinating activity
Hyp: Hydroxyproline
JC-1: 5,5′,6,6′-tetrachloro-1,1′,3,’-tetraethylbenzimidazolylcarbocyanine iodide
*S. integrifolium*: *Solanum integrifolium*
Sf: *Spodoptera frugiperda*
