# Supplementary material for: Functional characterization of chitin-binding lectin from Solanum integrifolium containing anti-fungal and insecticidal activities
Source: BMC Plant Biol. 2018 Jan 3;18:3. doi: 10.1186/s12870-017-1222-0 (PMC5751800; doi:10.1186/s12870-017-1222-0)
Supplement: Supplementary file 1 — Tryptic digested peptide sequences identified by MS. Table S2. Primer sequences used in this study. Table S3. The activity of CBL in various metal ions. Table S4. In vitro inhibition effect of CBL on fungus. (PDF 120 kb) [file 12870_2017_1222_MOESM1_ESM.pdf]

Supplement Table I Tryptic digested peptide sequences identified by MS

mass	position	peptide sequence
2891.4977	139-168	SPPPPSPPPPSPSPPPPSPP PPSPSPPPPK
2268.0561	111-133	DGGGCQSKPGAQSLEGY GTS LQK
1777.9112	53-69	TIQGQSATTALTMEVAR
1283.6156	44-52	IFOLIUMMK
1236.6809	169-178	IHFMFISTIK
1230.5538	74-83	HFFDYGSTTR
1176.5625	199-206	FFFWPNYR
1114.4656	180-191	CGLGSEFCGGGK
1036.4793	99-107	EISESNETK
723.2912	90-98	CGMGGGGGK
616.3664	192-196	NELIK
575.3035	134-138	EIEGK
507.2344	84-87	NSMR

Supplement Table II Primer sequences used in this study

**Primers**	**Sequence**
Degenerated primers for PCR cloning	
5-F1	atg aar can ath car ggn car wsn
5-F2	gcn can can gcn ytn can atg gar
5-F3	gtn gcn mgn gtn car gcn trr
3-R1	aar aay gar ytn ath aar
3-R2	tty aar tty tty tty tgg
3-R3	ccn aay tay mgn ytn
Sequence primers used for cDNA cloning	
5-F4	atg aaa acc atc caa ggc
3-R4	aag tcg ata att ggg cca

Supplement Table III The activity of CBL in various metal ions

**Metal ions**	**Residual activity (%)**
$CaCl_2$	116±7
$CuCl_2$	72±2
$FeCl_3$	101±5.5
$MgCl_2$	95±2.5
$MnCl_2$	96±1.2
$ZnCl_2$	98±0.6
EDTA	85±2.6

Supplement Table IV *In vitro* inhibition effect of CBL on fungus

Organism	Diameter of zone of inhibition (mm)		
	Crude extract (0.5 mg/mL)	Purified natural CBL (0.5 mg/mL)	Recombinant CBL (0.5 mg/mL)
C. gloeosporioides	0	2	8
R. solani	6	10	12

The boiled CBL was used as negative control.
