# Supplementary material for: Functional characterization of chitin-binding lectin from Solanum integrifolium containing anti-fungal and insecticidal activities
Source: BMC Plant Biol. 2018 Jan 3;18:3. doi: 10.1186/s12870-017-1222-0 (PMC5751800; doi:10.1186/s12870-017-1222-0)

Supplement Figure 1 The multiple alignment of LEL, STL and DSA to CBL

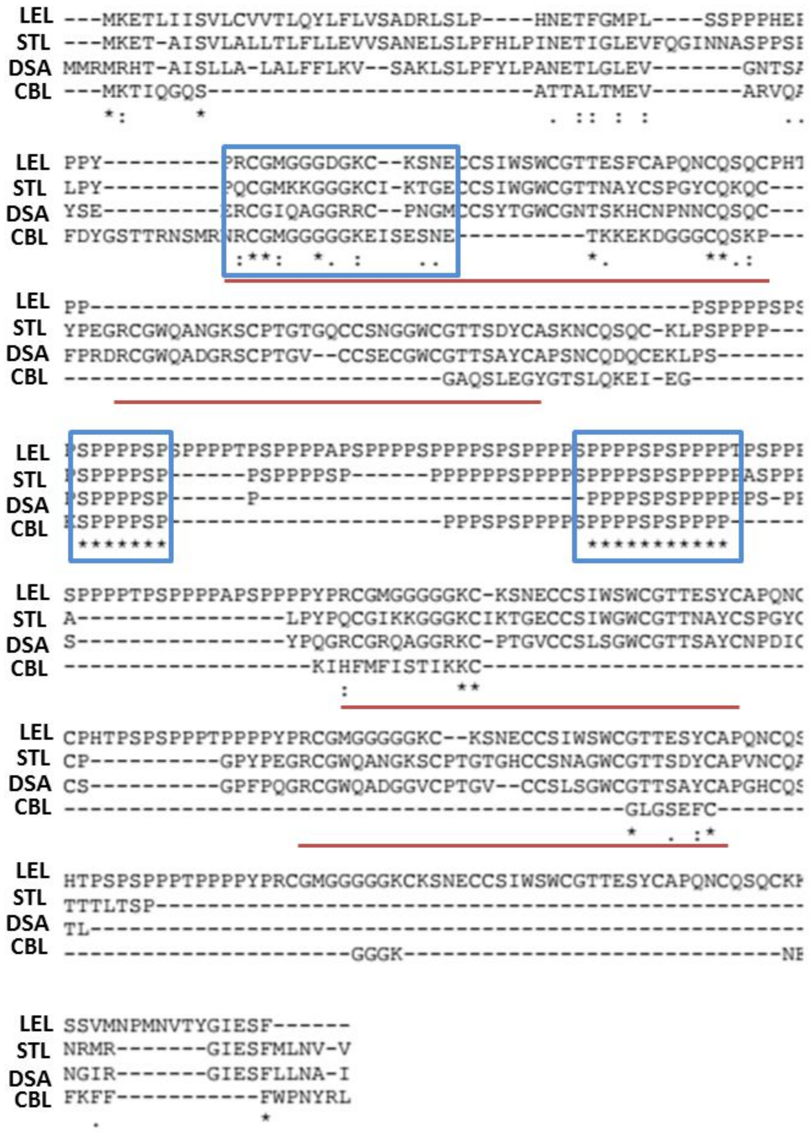

* indicated consensus sequences in four chitin binding proteins. High sequence similarity regions are indicated in blue.

Supplement Figure 2 The effect of temperature on CBL

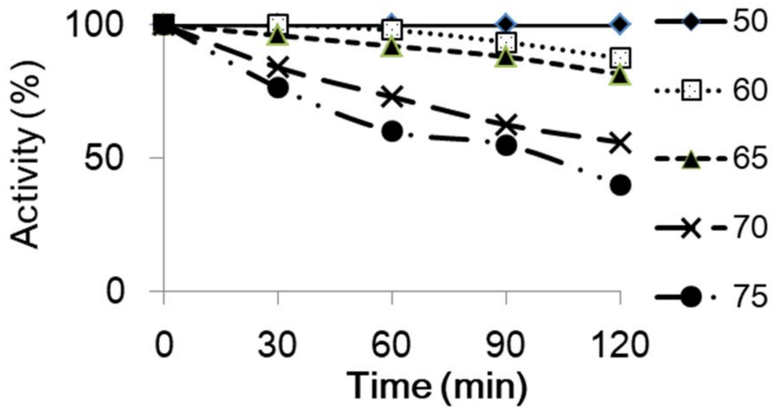

Supplement Figure 3 The effect of pH on CBL

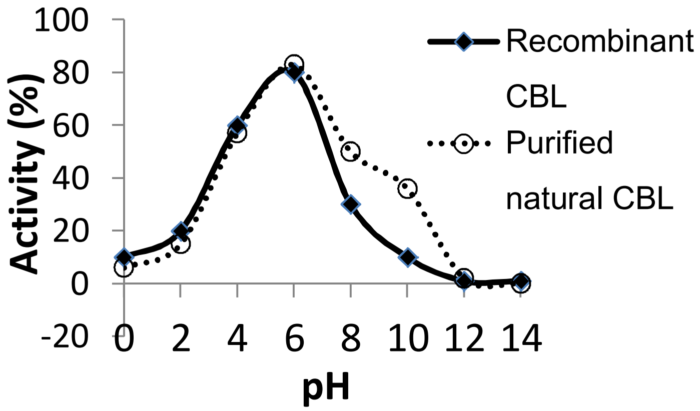

Supplement: Supplementary file 2 — The multiple alignment of LEL, STL and DSA to CBL. Multiple alignments were performed using MAFFT (http://www.ebi.ac.uk/Tools/msa/mafft/) under default settings (gaps and matches were equally weighted). Figure S2. The effect of temperature on CBL. The lectin was preheated with 50, 60, 65, 70, or 75 °C for different durations. Figure S3. The effect of pH on CBL. The lectin was incubated in the solutions of pH 0 to 14 at RT. Results are representative of >3 independent experiments. (PDF 341 kb) [file 12870_2017_1222_MOESM2_ESM.pdf]
